# Rapid parallel evolution overcomes global honey bee parasite

**DOI:** 10.1038/s41598-018-26001-7

**Published:** 2018-05-16

**Authors:** Melissa Oddie, Ralph Büchler, Bjørn Dahle, Marin Kovacic, Yves Le Conte, Barbara Locke, Joachim R. de Miranda, Fanny Mondet, Peter Neumann

**Affiliations:** 1 Institute of Bee Health, Vetsuisse Faculty, University of Bern, Bern, Switzerland; 2 LLH Bee Institute, Erlenstr, 9, 35274 Kirchhain, Germany; 3 Norwegian Beekeepers Association, Dyrskuev, 20, NO-2040 Kløfta, Norway; 4 Department of Animal and Aquacultural Sciences, Norwegian University of Life Sciences, PO Box 5003 NMBU, NO-1432 Kløfta, Ås Norway; 5 J.J. Strossmayer University of Osijek, Faculty of Agriculture, 31000 Osijek, Croatia; 6 INRA, UR 406 Abeilles et Environnement, Avignon, France; 7 Department of Ecology, Swedish University of Agricultural Sciences, Uppsala, 750 07 Sweden; 8 Agroscope, Schwarzenburgstrasse 161, 3003 Bern, Switzerland

## Abstract

In eusocial insect colonies nestmates cooperate to combat parasites, a trait called social immunity. However, social immunity failed for Western honey bees (*Apis mellifera*) when the ectoparasitic mite *Varroa destructor* switched hosts from Eastern honey bees (*Apis cerana*). This mite has since become the most severe threat to *A*. *mellifera* world-wide. Despite this, some isolated *A*. *mellifera* populations are known to survive infestations by means of natural selection, largely by supressing mite reproduction, but the underlying mechanisms of this are poorly understood. Here, we show that a cost-effective social immunity mechanism has evolved rapidly and independently in four naturally *V*. *destructor*-surviving *A*. *mellifera* populations. Worker bees of all four ‘surviving’ populations uncapped/recapped worker brood cells more frequently and targeted mite-infested cells more effectively than workers in local susceptible colonies. Direct experiments confirmed the ability of uncapping/recapping to reduce mite reproductive success without sacrificing nestmates. Our results provide striking evidence that honey bees can overcome exotic parasites with simple qualitative and quantitative adaptive shifts in behaviour. Due to rapid, parallel evolution in four host populations this appears to be a key mechanism explaining survival of mite infested colonies.

## Introduction

Eusocial insect colonies can be regarded as superorganisms in which cooperating individuals of overlapping generations are analogous to cells in a multicellular organism^1,2^. Such cooperation includes reproductive division of labour, brood care and parasite defence via social immunity^3–5^. Social immunity involves those behavioural, physiological or organisational traits that enhance colony health, irrespective of their consequences for the health of individual bees. It is a successful strategy that has contributed to the evolution of sociality in many insect species^6^ since the sacrifice and removal of infected individuals from the colony forces the parasite to hedge its virulence^7^ by either reducing its reproductive success or its transmission of pathogens, and thus pursue a more co-adaptive relationship with its host. For the host therefore, the colony-level benefits of social immunity normally outweigh the costs in terms of lost individuals^4,8^, especially for well-established, co-adapted pathogens. However, the host may require a re-calibration of the individual and social defences if it is to survive a novel parasite. The power of social immunity is such that this re-calibration can be achieved quickly, through simple shifts in behavioural patterns whose beneficial effects are amplified through the social structure and population turnover^9^. This dynamic may have a role to play in the case of the ectoparasitic mite *Varroa destructor*, that switched hosts from the Eastern honey bee (*Apis cerana*) to the Western honey bee (*Apis mellifera*) within the last century^10^. The mite is now near-ubiquitous in *A*. *mellifera* populations globally^10^ and though African honey bee (*A*. *m*. *scutellata*) hybrids are known to be resistant^3,11,12^ it is the primary biological cause for major colony losses of European honey bees worldwide^10,13–15^. This dramatic impact is induced mainly by mite-transmitted viruses, the most destructive being variants of Deformed Wing Virus (DWV)^16–18^, that increase winter mortality in the bees and can cause colonies to fail in approximately two years^10,13,16^. Nevertheless, some populations of European honey bee in *V*. *destructor*-positive regions have recently been found surviving mite infestations naturally without treatment for more than 17 years^3,19–23^. These populations (which will from here on be referred to as ‘surviving’ to imply naturally surviving *V*. *destructor* infestations without treatments) originated from initially mite-susceptible stocks and over the course of several generations, achieved survivability^3,19–23^. It is known that regular treatments by beekeepers limit honey bee natural selection^24^, and since *V*. *destructor* is a recent invasion^10,25^ this indicates a rapid host adaptation in all of these populations near-simultaneously. Previous studies have revealed that mite reproductive success is reduced in these populations to a point that permits colony survival^22,23^. However, at present there is no clear answer as to the cause of reduced mite reproduction in such surviving bees, be they naturally-adapted or selectively bred^3,26^.

**Figure 1:**
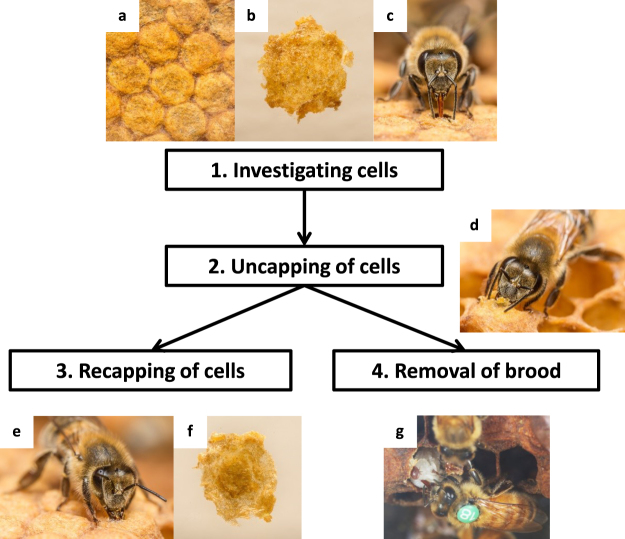
An ethogram that demonstrates the behavioural sequence involved in the social immunity of adult worker honey bees targeting mite-infested sealed brood cells. (**a**) Top view of worker brood cells, (**b**). Underside of an intact, worker cell capping that is completely covered by the glossy larval silk cocoon. The ethogram is comprisesd of four stages: 1. Investigating cells: workers scrutinize the wax capping of cells with their tongue and antennae (**c**). 2. Uncapping of cells: workers use their mandibles to remove the wax capping of cells (**d**). These first two stages are always performed in the same sequence. A major transition occurs after stage 2, where workers can decide between the following two stages. 3. Recapping of cells: workers use wax gland secretions and their mandibles to recap the cells (**e**), resulting in the underside of a recapped cell displaying a notable dark hole with a visually matte wax (**f**). 4. Removal of brood: workers pull out and discard the brood from mite-infested cells (**g**). The entire behavioural sequence is flexible due to decision making by multiple workers involved. The sequence can stop after stage 1. (i.e. cells are investigated, but not uncapped), or after stage 2. (i.e. uncapped cells can be recapped or not) and both uncapping and recapping can be only partly performed (i.e. only a part of the capping is uncapped and/or recapped). Photos in c, d & e © Anders Lindström.

There are two stages at which social immunity can act on mites: when they are wandering, actively feeding on adult bees or during the reproductive phase, when they are sealed in host brood cells^27^. Adult workers remove mites from themselves and/or nestmates through grooming and elevated levels of grooming have been shown to reduce mite infestations in *A*. *mellifera*^28^. It is however, expressed at low frequencies in the naturally-surviving honey bee populations investigated so far^3,22,23,29^ and does not contribute to a reduced mite reproductive success. Social immunity targeting brood cells (Fig. 1) includes removal of mite-infested brood with a notable bias for targeting cells with reproducing mites, a behaviour defined within varroa sensitive hygiene (VSH)^27,30^. This can reduce infestation rates by decreasing the proportion of successfully reproducing mites in the colony^30,31^. However, worker brood removal is only sustainable up to a point, provided the colony has sufficient resources to replace it. If workers are lost at a faster rate than they are being replaced, the colony enters a negative spiral and becomes terminal^7,10^. Even small losses may affect competitive ability. A less-costly solution is more likely to be favoured by natural selection as it would reduce mortality risk and increase colony competitiveness^10,13^. Since *V*. *destructor* is sensitive to subtle shifts in host-derived kairomones, temperature and humidity^32–34^, the simple opening of brood cells may be sufficient to impair mite reproduction. Such uncapping of sealed brood cells without removal, followed by their recapping (recapping, Fig. 1), is common in all honey bee populations where it has been specifically investigated^30,35–37^ and it is of a low cost for the colony since no brood is sacrificed in the action. Video recordings^38^ confirm the uncapping (Fig. 1d) and recapping (Fig. 1e) behaviour as well as the tell-tale signs it leaves on the cell cap (Fig. 1f). A possible association between recapping and reduced mite reproductive success has been reported from a population bred for VSH^31^. However, the presence and potential impact of recapping on mite reproductive success has not yet been investigated in those honey bee populations that survive mite infestations by means of natural selection. If recapping enhances honey bee colony fitness by reducing mite reproductive success, then we can expect adaptive differences in this behaviour in surviving populations subjected to natural selection: more frequent recapping and an enhanced targeting of mite-infested cells, compared to sympatric, susceptible control populations.

Here we conducted a survey to investigate the frequency of recapping, the selective targeting of mite-infested cells and its effect on the proportion of successfully reproducing mites in four naturally-surviving honey bee populations: two in France, one in Norway and one in Sweden, compared to local non-adapted bee populations. Cells were opened and their contents recorded, including the developmental stages of the honey bee pupae and of all the mites. The numbers of infested/opened cells examined in each population were as follows: France-Avignon (686/23,362); France-Sarthe (726/20,954); Norway (833/3,430); Sweden (116/868). Uncapping worker bees have been known to target infested cells with reproducing mites^31^. This introduces a selection bias where cells with non-reproductive mites are ignored (not uncapped). To account for this potential bias of the bees, we complemented the recapping survey of the naturally-surviving populations with an artificial uncapping experiment (29 brood samples with in total 1905 single infested cells, 769 uncapped cells and 1136 control cells). The experiment was designed to study the direct impact of cell uncapping on mite reproductive success without any selection bias by the adult bees, thereby complementing the correlative survey with a causation test for recapping as a mechanism for reducing mite reproductive success.

## Results

### Mite reproductive success

**Figure 2:**
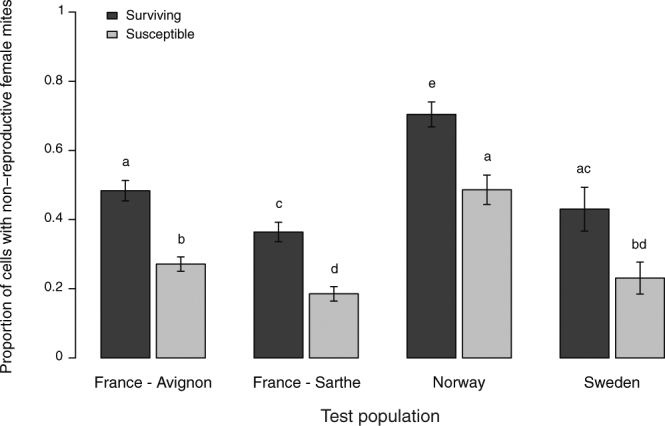
Adjusted mean proportions (+/−SE) of capped brood cells infested with a single mite foundress, *V*. *destructor*, that displayed non-reproduction in local surviving and susceptible honey bee colonies, *A*. *mellifera*, in Norway, Sweden and France (Avignon and Sarthe). The proportions of non-reproducing mites were significantly and consistently higher in surviving colonies compared to susceptible ones (GLMM, n = 74 colonies, see Table 1, Supplementary Information Table 3). Different letters indicate significant differences between groups (p < 0.05). There were significant differences between test populations (GLMM: χ^2^ = 48.72, p < 0.001, n = 74).

The survey data show that proportions of non-reproducing female mites were significantly increased in all naturally-surviving honey bee populations compared to local susceptible colonies, reducing reproductive success by 10–30% (Fig. 2, Table 1, χ^2^ = 51.14, p < 0.001). Of all the populations investigated, the Norwegian mite-surviving population had the highest proportion of non-reproductive female mites, with an average number of viable female offspring at 0.84 per foundress (Fig. 2, Table 1, Supp. information Table 3).

**Table 1:** Factors for the reproductive parameters of ectoparasitic mites, *V*. *destructor*, within surviving and susceptible host populations of European honey bee subspecies, *A*. *mellifera*.

Response variable	Explanatory variable	*n*	DF	χ^2^	*P* Value
Rate of non-reproduction	Population	74	3	48.72	1.49 e 10^−10^
	Resistance level		1	51.14	8.58 e 10^−13^
	Brood stage		1	1.19	0.28

Rate of non-reproduction was counted as the number of infested cells that failed to produce viable female offspring. Population is described as groups of independent colonies sampled in different regions: France (Avignon, Sarthe), Norway and Sweden. Resistance level is described as populations of surviving or susceptible bees within the same region of study. Brood stage was the mean estimated age of the capped brood examined per colony. A GLMM was fitted to the data, with colony as the individual and colony ID as a random effect. A binomial distribution with a response vector was used to assess the rate of non-reproduction.

### Recapping

**Figure 3:**
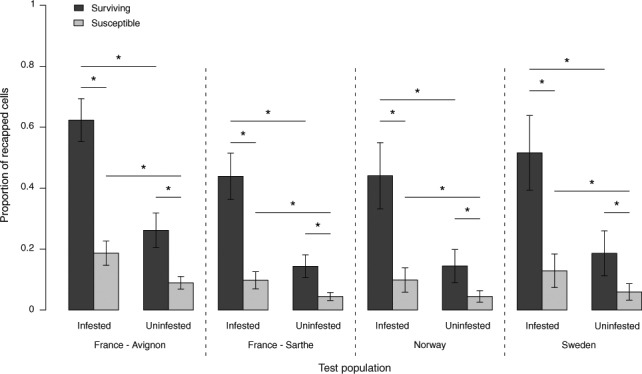
Adjusted mean proportions (+/−SE) of naturally recapped worker brood cells, infested or uninfested with *V*. *destructor* mites, in local colonies of surviving and susceptible European honey bees, *A*. *mellifera*, in Norway, Sweden and France (Avignon and Sarthe) (n = 74 colonies). In all populations, the surviving colonies showed both significantly higher frequencies of recapping and a significant targeting of mite-infested cells compared to the local susceptible colonies (GLMM, see Table 2, Supplementary Information Table 4). Asterisks indicate significant differences between groups (p < 0.05). There were no significant differences between test populations (GLMM: χ^2^ = 5.43, p = 0.143, n = 74).

**Figure 4:**
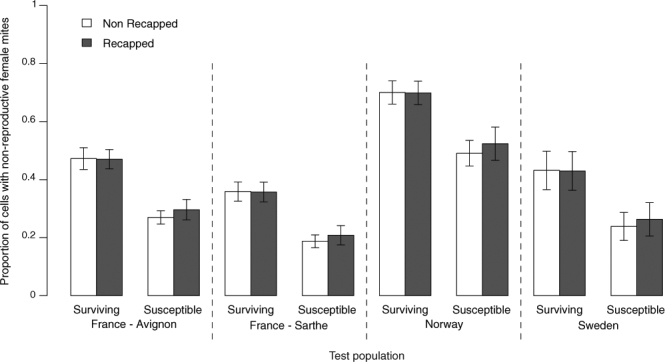
Proportions of non-reproductive mites, *V*. *destructor*, in naturally recapped vs. non-recapped worker brood cells of the four surviving *A*. *mellifera* populations and local-susceptible populations. Adjusted mean proportions (+/−SE) of non-reproductive mites in naturally uncapped/recapped worker brood cells vs non-targeted worker brood cells, of the four surviving and local-susceptible *A*. *mellifera* populations (n = 74 colonies). There were significant differences between test populations (GLMM: χ^2^ = 50.84, p < 0.001, n = 74).

**Figure 5:**
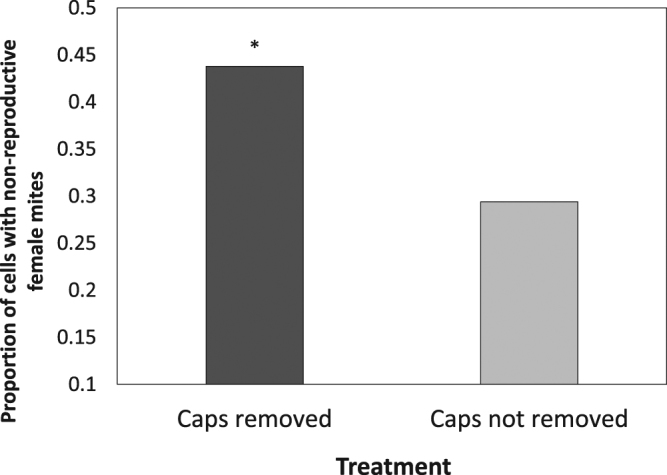
Proportions of non-reproductive mites, *V*. *destructor*, in single infested, artificially-uncapped honey bee, *A*. *mellifera*, worker brood cells. Significantly more mites did not reproduce when the cell caps were experimentally removed (GLMM: χ^2^ = 50.231 p < 0.001, n = 1905 cells).

The frequency of recapping and the specific targeting of mite-infested cells were significantly higher in surviving colonies than in the local susceptible controls (Fig. 3, Table 2, Supp. information Table 4, Frequency: χ^2^ = 23.11, P < 0.001, Targeting: χ^2^ = 34.54, p < 0.001). The recapping rates among surviving populations were not statistically different from one another and the rates among susceptible populations were also comparable (Table S3). There was no significant difference between the recapped and undisturbed cells in terms of the proportion of non-reproductive mites in surviving or local-susceptible populations (Fig. 4). In contrast, the experimental uncapping (as it was indiscriminate) showed that recapping does significantly reduce mite reproductive success (Fig. 5, χ^2^ = 50.231 p < 0.001).

**Table 2:** Factors for the frequency of recapping behaviour within surviving and susceptible populations of European honey bee subspecies, *A*. *mellifera*.

Response variable	Explanatory variable	*n*	DF	χ^2^	*P* Value
Recapping	Population	74	3	5.43	0.14
	Resistance level		1	23.11	1.52 e 10^−6^
	Cell type		1	439.8	<2.2 e 10^−16^
	Brood stage		1	4.96	0.026
	Cell type × Resistance level		1	34.54	4.19 e 10^−9^

Recapping was counted as the number of cells that had been recapped amongst the dissected cells in each colony. Population is described as groups of independent colonies sampled in different regions: France (Avignon, Sarthe), Norway and Sweden. Resistance level is described as populations of surviving or susceptible bees within the same region of study. Cell type is described as the status of infested or non-infested of brood cells. Brood stage was the mean estimated age of the capped brood examined per colony. A GLMM was fitted to the data, with colony as the individual, and the colony ID as a random effect. A binomial distribution with a response vector was used to assess the rate of recapping.

## Discussion

The results of our survey clearly show that recapping is both more frequent and targeted towards mite-infested cells in all of the investigated populations surviving by means of natural selection when compared to local susceptible controls. *V*. *destructor* rates of non-reproduction were consistently higher in all surviving populations, confirming the results of previous studies^22,23^ and displaying rates of reduced reproductive success not unlike other known surviving populations of European and Africanized honey bees^3,12,39^. Reproductive rates were about 0.84 offspring per foundress in the Norwegian population, which is in line with previous studies performed on naturally varroa-surviving Africanised honeybees^11^ (0.79) and European honey bees on a Brazilian Island^40^ (0.54). These results concur with observations in varroa-surviving African and Africanized honey bee populations that display a ‘bald brood’ pattern with large amounts of uncapped brood cells^11,35,38,39^. This also supports the idea that uncapping/recapping by adult bees is a behavioural mechanism mitigating mite infestations. Mite offspring mortality was not examined in detail in this study. However, it has previously been found to be higher in recapped cells^31^ further supporting recapping as an effective mechanism promoting *V*. *destructor*-survivability. The survey showed no differences in mite reproductive success between recapped and untouched cells, which is similar to honey bees bred for VSH^31^. Intuitively, one would expect that if recapping had a direct effect on mite reproductive success there would be an increase in the number of non-reproductive mites in the cells that had been recapped. However, with the knowledge that bees are selective in their targeting of infested cells^31^; if uncapping workers focus their efforts on cells with the most mite reproduction and ignore cells with a lower reproduction, the observed rates of reproduction in recapped and untouched cells may become similar, but through different mechanisms. Recapped cells would have a lower mite reproductive rate due to the direct effect of recapping, while cells with naturally low or non-reproductive mites would be ignored by uncapping bees, and consequently be underrepresented in the recapped cohort. Therefore, the absence of differences in mite reproductive success between recapped and untouched cells is uninformative due to the selection bias introduced by the bees. However, the experimental uncapping of brood cells showed that recapping can reduce mite reproductive success directly, without sacrificing nestmates. The efficacy of recapping as a natural mechanism for reducing mite reproductive success could only be shown by combining the survey data with the experimental uncapping, since the latter excluded any biased targeting of mite-infested cells. Our experiment demonstrated that recapping behaviour can cause reduced colony-level mite reproductive success and is not only correlated with it.

The data therefore provide clear evidence that recapping is a cost-effective social immunity mechanism that helps, in part, explain the natural survival of European honeybees with unmanaged *V*. *destructor* infestations. We cannot exclude that the removal of infested brood^30^ and other factors may have also contributed to the natural mite-survival of these populations. Indeed, most likely the phenotype of naturally *V*. *destructor*-surviving honey bee colonies is determined by a series of host traits^19,28–30,41,42^, local genotype-environment interactions^43^, pathogen variation^44^, resource availability^45^ and beekeeping management^24^. However, due to its rapid and parallel evolution in the four studied surviving populations and the significant impact on mite reproductive success, recapping appears to be a common and previously-overlooked key mechanism for colony survival. Brood removal, though potentially linked to recapping is unlikely to be a primary mechanism since the loss of individuals would compromise colony competitiveness^46^. In addition, the Norwegian population under study is known to display no increased rates of brood removal^23^, indicating that, at least for that population, it was not a prominent selected mechanism for survival. In sharp contrast, recapping involves only the time and energy to manipulate wax cappings. It therefore appears to be a much more cost-effective mechanism than brood removal to achieve similar ends. Mite reproduction need not be terminated completely, only reduced to affect population growth sufficiently to ensure colony survival. The timing in which mite-susceptible bees develop a surviving colony phenotype can be currently estimated to a minimum of 17 years^3,22,23,29^, though this period may indeed be shorter. The speed of the adaptation can be explained in four points: (1) The trait very likely stems from a preadaptation as uncapping/recapping behaviour has been recorded in all investigated honey bee populations so far^23,30,35–37,40^, (2) elevated recapping and mite targeting can be explained by simple shifts in worker behavioural thresholds^47^. Behavioural shifts in a social immunity repertoire can be optimized incrementally with considerable impact at the colony level. (3) The penalty for uncapping a non-infested cell is not lethal to the brood, giving inevitable errors a much softer impact, and (4) the behavioural sequence targeting/uncapping/investigating/removal or recapping (Fig. 1) can be performed by different workers^48^, which offers ample opportunity for independent, non-linked adaptive shifts. It is likely that recapping is a common trait in many if not all honey bee populations and that the ancestors of our current surviving populations were individual colonies that were displaying both high recapping frequency and sensitivity to brood health.

In light of our findings and the ubiquity of recapping in honey bee populations^31,35–37^, this behavioural sequence appears to be an integral part of brood care and pathogen defence in *A*. *mellifera*. Recapping would in theory have similar health benefits for other eusocial insects with sealed brood cells, such as the Asian honey bees, bumblebees, stingless bees and social wasps^49^, however, this remains a testable hypothesis. Despite comparatively long generation intervals^50^, it is possible for eusocial insect colonies to evolve low-cost social immunity traits via simple qualitative and quantitative adaptive shifts in worker behaviour. Such behavioural adaptability of workers can explain the general success of eusocial insects^6^, by fostering colony-level homeostasis during periods of rapid environmental change. Aiming at sustainable global apiculture, it appears prudent to employ evolutionary thinking to manage infectious diseases by taking advantage of efficient mechanisms favoured by natural selection^23,24^.

## Methods

### Survey: Natural recapping behaviour

#### Locations and Timing

Colonies from the four independent surviving honey bee (*A*. *mellifera*) populations had been managed without mite treatments for >17 years (Sweden: 18 years^19^, France: 23 years^20^ and Norway: <20 years^23^). Similar numbers of surviving colonies and local susceptible controls were surveyed in each location. All studies were conducted between late local summer to fall (August to September), when local *V*. *destructor* mite populations are generally at their highest. The queens of all colonies in the study were naturally mated. Mite reproductive success and recapping behaviour were measured in local surviving and susceptible colonies in all four populations using similar methods described below.

**Norway**: Experiments were conducted with surviving and susceptible *A*. *m*. *carnica* colonies in separate apiaries 60 km apart near Oslo between August and September 2015, with the susceptible controls located within an apicultural conservation area. Five susceptible and five surviving colonies were randomly chosen from their respective apiaries. Due to the scarcity of mites in the surviving colonies, donor brood frames from ten highly infested susceptible colonies 50 km away from both experimental apiaries were inserted into the experimental surviving colonies for estimating the various parameters.

**Sweden**: All experimental colonies were located and examined in Uppsala. The susceptible control colonies were derived from a local Buckfast population that was geographically isolated from the mite-surviving bee population on the island of Gotland. The mite-surviving colonies used in this study contained queens reared and free-mated within the isolated population on Gotland before being introduced into the experimental colonies in Uppsala. Four surviving and four susceptible colonies were randomly chosen to record recapping frequencies and mite reproductive success in late September 2016.

**France**: Two distinct surviving and susceptible populations were included and kept at the INRA apiaries in Avignon, as well as in Sarthe. All observations took place between August and September in 2015 and 2016. Susceptible queens were of a mixed local *A*. *m*. *carnica - A*. *m*. *ligustica* or Buckfast stock. In Avignon, 12 surviving and 21 susceptible colonies were randomly chosen from three separate apiaries within the region. Due to the proximity of surviving and susceptible colonies, queens of the surviving stock had the possibility of hybridizing with susceptible males so the surviving trait could only be guaranteed in the maternal lineage. In Sarthe, 12 surviving and 11 susceptible colonies were randomly chosen from three separate apiaries within the region. Susceptible colonies were mated in apiaries located within several kilometres from the apiaries where the surviving colonies are residential.

**Control populations**: Local adaptation is known to be a significant factor in the health and success of honey bees, often more important to consider than genetic origin^43^. All controls were chosen for their geographical sympatry with the surviving populations. Control colonies were known to require regular treatment against *V*. *destructor* or else suffer severe losses.

#### Identifying recapped cells

The recapping behaviour can be easily detected as a hole in the spun cocoon of the pupated larva ranging in size from one mm to the entire area of the cap. The hole is subsequently covered over with wax by the adult bees. This hole can be seen as a dark, matte spot on the underside of the cell cap distinct from the glossy coating of the cocoon. Cells were identified following earlier protocols^27,31^. The sealed worker brood cells (Fig. 1a) were opened carefully using forceps so the cap was preserved as a whole. The cap was then placed inverted under a dissecting microscope and examined carefully. If the cocoon was intact (Fig. 1b), the cell was marked as ‘untouched’, if there was a notable hole in the silk cocoon (Fig. 1f) the cell was marked as ‘recapped’ Each cell opened was given a binary score of ‘infested’ or ‘uninfested’.

#### Measuring mite reproductive success

Once all contents had been removed from the cell, the developmental stages of the bee brood and of all mites were noted^22,51,52^. Only single foundress-infested cells were considered in these analyses. The measure of foundress reproduction was based on the ‘effective reproduction rate’, which is interpreted as the potential number of viable female offspring per foundress^52^. Offspring were only considered viable if they were of an adequate stage to survive upon bee emergence and if at least one male was present within the cell^52,53^. All cells that did not have daughter mites meeting these requirements were given a value of zero; all cells that met these requirements were given a value of one. Proportions of successfully-reproducing cells were taken by colony. If no evidence of nymphs or eggs could be found, the foundress contained in the cell was marked as non-reproductive and also given a value of zero. Brood estimated to be <170 h old was not considered.

#### Frame preparation and cell dissection

A range of 150–300 cells were dissected on each frame with infested cell numbers ranging from 10–50 per colony. All colonies contributed a single frame to the study while the Norwegian colonies contributed two frames each. For all populations brood infestation rates, recapping frequencies and mite reproductive success were measured using established methods^22,31,52^. Cells were carefully opened using fine forceps and the pupae were gently removed. Mites clinging to the body were brushed off with a small paint brush. The cell interior was also brushed carefully to extract but not damage the younger, softer-bodied mites and eggs. All contents of the cells was examined carefully under dissecting microscopes^22,52^. Within the Swedish and French populations, frames were examined fresh from the colonies. In Norwegian populations, brood frames were transferred to surviving and susceptible receiver colonies and placed in the centre of the brood box just after the majority of the brood had been capped. The frames were mapped and photographed prior to the introduction and ∼10 days afterwards, when the majority of the brood was close to adult emergence^50^. Frames were then frozen prior for dissections.

### Experimental recapping

The experiment was performed in an apiary close to Kirchhain, Germany. Five brood samples were collected from four colonies in August 2013 and another 24 from four colonies in August 2014. While the sampled colonies had freely laying queens in 2013, the queens were consecutively caged for two days on individual combs (trapping combs) in 2014, in order to produce a defined and uniform age cohort of brood. Brood stage was accounted for in statistical modelling.

Combs with sealed worker brood were uncapped by placing strips of linen soaked in molten wax on the caps and removing them again after adhesion^54^. If the cappings of individual cells were not fully removed by the wax strips they were fully opened with forceps. Therefore, all cells in the treated area were uncapped. The uncapped cells (treatment) and a similar number of untouched sealed brood cells (control) were marked on plastic sheets. Immediately after treatment the combs were returned to their original position in the brood nest of their maternal hives and were recapped by the adult bees. The combs remained in their colonies until the bees developed close to natural emergence^50^. Therefore, the age that the cells were uncapped varied over almost a week. This reflects natural conditions because it is very likely that recapping can occur at any point from capping to emergence and may even occur multiple times in some cells. Depending on the age of the brood at the time of treatment, the combs were removed after 4–10 days and immediately transferred to a freezer (−18 °C) where they remained until dissections.

The individual brood cells were opened and inspected under a microscope with about 10-fold magnification. Only elder pupae with purple or black eyes (170+ hours post-capping)^50^ were considered and carefully checked for *V*. *destructor* infestations and reproductive success. The brood infestation rate was calculated as the number of infested cells divided by the total number of evaluated cells. Cells infested by a single mite foundress were classified as reproductive if at least one female deutonymph or an adult daughter mite was present. Otherwise, they were classified as non-reproductive. The rate of non-reproductive cells was calculated as the number of non-reproductive cells divided by the total number of single-infested cells.

### Statistical analyses

All statistical analyses and figures were generated in the R environment (Version 3.3.1)^55^. For the description of recapping and non-reproduction in the four European populations, bee colonies were considered as the statistical individual, since the traits of interest are expressed at the colony level. Due to the nature of the experimental designs, analyses were performed using general linear mixed-effect models (GLMEM – package *lme4*)^56^. The proportion of recapped or non-reproducing cells was represented by a response factor and a binomial distribution (link: logit). Fixed explanatory variables included the population (France Avignon, France Sarthe, Norway, Sweden), the resistance level (surviving or susceptible), the infestation status (infested or not – for recapping only) and the brood stage, and the colony ID was considered as a random factor. Residuals and over-dispersion were analysed with the *RVAideMemoire* package^57^. Pairwise comparison between groups and estimations of the adjusted means were performed with the *lsmeans* package^58^.

### Availability of materials and data

All collected data are provided in the supplementary information or available upon request directed to corresponding authors.

## Electronic supplementary material

Supplementary Information

Dataset 1 Avignon fecund

Dataset 2 Avignon recap

Dataset 3 Experiment recap

Dataset 4 Norway fecund

Dataset 5 Norway recap

Dataset 6 Sarthe fecund

Dataset 7 Sarthe recap

Dataset 8 Sweden fecund

Dataset 9 Sweden recap
