# Supplementary material for: Rapid parallel evolution overcomes global honey bee parasite
Source: Sci Rep. 2018 May 16;8:7704. doi: 10.1038/s41598-018-26001-7 (PMC5955925; doi:10.1038/s41598-018-26001-7)
Supplement: Supplementary file 1 — Supplementary Information [file 41598_2018_26001_MOESM1_ESM.docx]

**Supplementary Information**

**Rapid parallel evolution**

**overcomes global honey bee parasite**

Melissa Oddie^1^*, Ralph Büchler^2^*, Bjørn Dahle^3,4^, Marin Kovacic^2,5^, Yves Le Conte^6^, Barbara Locke^7^, Joachim R. de Miranda^7^, Fanny Mondet^6^, Peter Neumann^1,8^

* Equal contributions

1. Institute of Bee Health, Vetsuisse Faculty, University of Bern, Bern, Switzerland

2. LLH Bee Institute, Erlenstr. 9, 35274 Kirchhain, Germany

3. Norwegian Beekeepers Association, Dyrskuev. 20, NO-2040 Kløfta, Norway

4. Department of Animal and Aquacultural Sciences, Norwegian University of Life Sciences, PO Box 5003 NMBU, NO-1432 Ås, Norway

5. J.J. Strossmayer University of Osijek, Faculty of Agriculture, 31000 Osijek, Croatia

6. INRA, UR 406 Abeilles et Environnement, Avignon, France,

7. Department of Ecology, Swedish University of Agricultural Sciences, Uppsala 750 07, Sweden

8. Agroscope, Schwarzenburgstrasse 161, 3003 Bern, Switzerland

# corresponding authors:

1) Melissa Oddie (email: melissa.oddie@vetsuisse.unibe.ch)

2) Ralph Büchler (email: ralph.buechler@llh.hessen.de)

**Supplementary Information Table 3 |** Pairwise comparisons of the proportion of cells with non-reproducing female mites in the four populations of European honey bee subspecies, *A. mellifera*.

| **Comparison** | **Estimate** | **Z-value** | **P-value** |
| --- | --- | --- | --- |
| **France Avignon – France Sarthe** | 0.4692651 | 3.075 | **0.0113** |
| **France Avignon - Norway** | -0.9597505 | -5.062 | **<.0001** |
| France Avignon - Sweden | 0.1638179 | 0.603 | 0.9311 |
| **France Sarthe - Norway** | -1.4290155 | -7.103 | **<.0001** |
| France Sarthe - Sweden | -0.3054472 | -1.095 | 0.6925 |
| **Norway - Sweden** | 1.1235683 | 3.718 | **0.0012** |

Post-hoc pairwise comparisons are presented for each of the populations (Tukey method). Comparisons in bold indicate significant differences between groups (p < 0.05).

**Supplementary Information Table 4 |** Pairwise comparisons of the frequency of recapping behaviour within surviving and susceptible populations of European honey bee subspecies, *A. mellifera*.

| **Comparison** | **Estimate** | **Z-value** | **P-value** |
| --- | --- | --- | --- |
| Surviving, France Avignon - Susceptible, France Avignon | 1.630614659 | 5.331 | <.0001 |
| Surviving, France Avignon - Surviving, France Sarthe | 0.748283642 | 2.127 | 0.3975 |
| Surviving, France Avignon - Susceptible, France Sarthe | 2.378898301 | 4.818 | <.0001 |
| Surviving, France Avignon - Surviving, Norway | 0.740634323 | 1.557 | 0.7758 |
| Surviving, France Avignon - Susceptible, Norway | 2.371248982 | 4.026 | 0.0015 |
| Surviving, France Avignon - Surviving, Sweden | 0.439080709 | 0.851 | 0.9901 |
| Surviving, France Avignon - Susceptible, Sweden | 2.069695368 | 3.352 | 0.0183 |
| Susceptible, France Avignon - Surviving, France Sarthe | -0.882331017 | -2.019 | 0.4690 |
| Susceptible, France Avignon - Susceptible, France Sarthe | 0.748283642 | 2.127 | 0.3975 |
| Susceptible, France Avignon - Surviving, Norway | -0.889980336 | -1.646 | 0.7224 |
| Susceptible, France Avignon - Susceptible, Norway | 0.740634323 | 1.557 | 0.7758 |
| Susceptible, France Avignon - Surviving, Sweden | -1.191533950 | -2.047 | 0.4498 |
| Susceptible, France Avignon - Susceptible, Sweden | 0.439080709 | 0.851 | 0.9901 |
| Surviving, France Sarthe - Susceptible, France Sarthe | 1.630614659 | 5.331 | <.0001 |
| Surviving, France Sarthe - Surviving, Norway | -0.007649319 | -0.015 | 1.0000 |
| Surviving, France Sarthe - Susceptible, Norway | 1.622965340 | 2.780 | 0.0998 |
| Surviving, France Sarthe - Surviving, Sweden | -0.309202933 | -0.578 | 0.9991 |
| Surviving, France Sarthe - Susceptible, Sweden | 1.321411726 | 2.158 | 0.3776 |
| Susceptible, France Sarthe - Surviving, Norway | -1.638263978 | -2.814 | 0.0915 |
| Susceptible, France Sarthe - Susceptible, Norway | -0.007649319 | -0.015 | 1.0000 |
| Susceptible, France Sarthe - Surviving, Sweden | -1.939817592 | -3.126 | 0.0376 |
| Susceptible, France Sarthe - Susceptible, Sweden | -0.309202933 | -0.578 | 0.9991 |
| Surviving, Nor - Susceptible, Norway | 1.630614659 | 5.331 | <.0001 |
| Surviving, Nor - Surviving, Sweden | -0.301553614 | -0.488 | 0.9997 |
| Surviving, Nor - Susceptible, Sweden | 1.329061045 | 1.939 | 0.5235 |
| Susceptible, Nor - Surviving, Sweden | -1.932168273 | -2.784 | 0.0988 |
| Susceptible, Nor - Susceptible, Sweden | -0.301553614 | -0.488 | 0.9997 |
| Surviving, Sweden - Susceptible, Sweden | 1.630614659 | 5.331 | <.0001 |

Recapping was counted as the number of cells that had been recapped amongst the dissected cells in each colony. Population is described as groups of independent colonies sampled in different regions: France (Avignon, Sarthe), Norway, and Sweden. Resistance level is described as populations of surviving or susceptible bees within the same region of study. Post-hoc pairwise comparisons are presented for each level of resistance and for each of the populations (Tukey method).
